# Hypoparathyroidism and pseudohypoparathyroidism: etiology, laboratory features and complications

**DOI:** 10.1590/2359-3997000000221

**Published:** 2016-11-07

**Authors:** Maicon Piana Lopes, Breno S. Kliemann, Ileana Borsato Bini, Rodrigo Kulchetscki, Victor Borsani, Larissa Savi, Victoria Z. C. Borba, Carolina A. Moreira

**Affiliations:** 1 Universidade Federal do Paraná Curitiba PR Brasil Serviço de Endocrinologia e Metabologia do Paraná (SEMPR), Universidade Federal do Paraná (UFPR), Curitiba, PR, Brasil; 2 Departamento de Medicina Interna Universidade Federal do Paraná Curitiba PR Brasil Departamento de Medicina Interna da Universidade Federal do Paraná (UFPR), Curitiba, PR, Brasil; 3 Divisão de Histomorfometria Óssea Fundação Pró-Renal Curitiba PR Brasil Laboratório P. R .O., Divisão de Histomorfometria Óssea, Fundação Pró-Renal, Curitiba, PR, Brasil

**Keywords:** Hypoparathyroidism, renal complication, renal calcification, hypocalcemia, pseudohypoparathyroidism

## Abstract

**Objectives:**

To identify a clinical profile and laboratory findings of a cohort of hypoparathyroidism patients and determine the prevalence and predictors for renal abnormalities.

**Materials and methods:**

Data from medical records of five different visits were obtained, focusing on therapeutic doses of calcium and vitamin D, on laboratory tests and renal ultrasonography (USG).

**Results:**

Fifty-five patients were identified, 42 females and 13 males; mean age of 44.5 and average time of the disease of 11.2 years. The most frequent etiology was post-surgical. Levels of serum calcium and creatinine increased between the first and last visits (p < 0.001 and p < 0.05, respectively); and serum levels of phosphate decreased during the same period (p < 0.001). Out of the 55 patients, 40 had USG, and 10 (25%) presented with kidney calcifications. There was no significant difference in the amount of calcium and vitamin D doses among patients with kidney calcifications and others. No correlation between serum and urinary levels of calcium and the presence of calcification was found. Urinary calcium excretion in 24h was significantly higher in patients with kidney calcification (3.3 mg/kg/d) than in those without calcification (1.8 mg/kg/d) (p < 0.05).

**Conclusions:**

The reduction of hypocalcemia and hyperphosphatemia suggest an effectiveness of the treatment, and the increase in serum creatinine demonstrates an impairment of renal function during follow-up. Kidney calcifications were prevalent in this cohort, and higher urinary calcium excretion, even if still within the normal range, was associated with development of calcification. These findings suggest that lower rates of urinary calcium excretion should be aimed for in the management of hypoparathyroidism.

## INTRODUCTION

Hypoparathyroidism (HP) is a metabolic disorder caused either by deficient or absent production of the parathyroid hormone (PTH) by the parathyroid glands or by resistance to PTH in its target tissues (a condition called pseudo-HP) (1,2). HP is a rare disease, with an estimated prevalence of 37 cases per 100,000 inhabitants in the USA (3). In Brazil, studies about the disease itself and data about its epidemiology are lacking. The lower PTH production by the parathyroids may be induced by damage to the glands during surgical procedures of the anterior cervical region (as total thyroidectomy or parathyroidectomy), then being called post-surgical HP; or by autoimmune, infiltrative, genetic or irradiative causes. Physiologically, PTH acts in order to maintain serum levels of calcium and phosphate within the normal range (8.8 to 10.4 mg/dL and 2.5 to 4.5 mg/dL, respectively). Its mechanism of action includes stimulation of bone remodeling with release of calcium and phosphate from the skeleton, and stimulation of the 1α-renal hydroxylase – an essential enzyme for production of calcitriol, which is the active form of vitamin D and therefore important for calcium absorption and bone resorption. In addition, PTH stimulates calcium reabsorption and phosphate secretion in renal tubular cells (1,3,4).

HP is clinically characterized by hypocalcemia and hyperphosphatemia (5,6). The main manifestations of HP include fatigue, paresthesia, tetany, Trousseau and Chvostek signs, cramps, convulsion, hyperreflexia, and cardiac disturbances such as an enlargement of the QT interval on the ECG. All of these are consequences of the muscle hyperexcitability caused by extracellular hypocalcemia (2,5). Other described manifestations are soft tissue calcification, as in basal ganglia; psychiatric symptoms such as depression; cataract; and alopecia, among others (7,8).

HP is one of the few endocrine hyposecretory diseases whose treatment, in most countries, does not consist of administration of the defective hormone. Rather, it consists of supplementation with high doses of calcium and vitamin D analogues, such as calcitriol and cholecalciferol (9-11). Recently, renal complications have been linked to HP (9). In fact, as the activity of PTH in the renal tubules favoring calcium reabsorption is not repaired, the supplemented calcium is rapidly excreted in the urine. Furthermore, the doses of calcium and vitamin D are usually very high and hypercalciuria is often seen in these patients. Chronic hypercalciuria may lead to nephrocalcinosis, nephrolithiasis, and renal failure (12).

The aims of this study are to identify a clinical profile and laboratorial findings of patients with hypoparathyroidism and to determine the prevalence as well as the predictors for renal abnormalities.

## MATERIALS AND METHODS

### Selection of patients

This is a retrospective, analytical, and observational study. Data have come from patients of the Bone Clinic of the Division of Endocrinology at Serviço de Endocrinologia e Metabologia da Universidade Federal do Paraná (SEMPR). This study has the approval of the local Ethics Committee in Research on Human Beings.

An active search for patients with HP was performed through the hospital database. The exclusion criterions were disease duration of less than 6 months, which is defined as transient HP (14). Patients whom the cause of the parathyroid surgery were secondary hyperparathyroidism due long-standing renal failure were also excluded from this analyses.

### Data collection

Clinical features such as age, gender, HP etiology, age at diagnosis, duration of disease, and doses of treatment (calcium, calcitriol, and/or cholecalciferol) were collected and analyzed. Renal ultrasonography (USG) and cranial tomography (CT), when available, were analyzed to identify the presence of such calcifications as nephrolitiasis and nephrocalcinosis in the kidneys and basal ganglia in the brain. The mean and standard deviation (SD) of serum total calcium, phosphate, creatinine, and PTH as well as 24-hour urinary calcium excretion were collected based on the first and last visit of each patient, as well as on three intermediary visits (5 time points). For the measurement of total serum calcium, phosphorus and creatinine standard kits were used, and analysis was performed using automated spectrophotometric equipment (ADVIA 1650, Bayer, Leverkusen, Germany). Levels of iPTH were determined by chemiluminescence (DPC, Immulite 2000, Los Angeles, CA, USA). The mean doses along with the range (minimum and maximum) of calcium carbonate, calcitriol, and cholecalciferol used by the patients at the five time points were also calculated.

Cockroft-Gault formula (15) was used to calculate the estimated glomerular filtration rate (eGFR) of those patients with weight and creatinine available for the last visit.

Patients who had renal USG results available were divided into two groups – with and without renal calcification – and were compared with each other regarding their clinical and laboratory features as well as doses of calcium and vitamin D.

### Statistical analysis

Data were described as frequency and percentage or by mean and SD for qualitative and quantitative variables, respectively. The results of the first and last visit were analyzed. Student’s t-test for paired samples or nonparametric Wilcoxon test and/or binomial test was performed as appropriate for quantitative and qualitative variables. Spearman’s correlation coefficients were estimated to test the association of disease duration with the laboratory results. For the analysis between the groups with and without renal calcification, Student’s t-test for independent samples or nonparametric Mann-Whitney test was used for the quantitative variables, and Fisher’s exact test for the qualitative variables. Results with values of p < 0.05 have been considered as statistically significant. The data were analyzed using the computational software IBM SPSS Statistics v.20.0.

## RESULTS

Main clinical characteristics of the patients are presented in Table 1. Fifty-five patients were identified: 42 (76.4%) females and 13 (23.6%) males, with a mean age of 44.5 ± 19.3 years. Post-surgical HP was the etiology in 41 (74.5%) patients, while 5 (9.1%) had pseudo-HP and 9 (16.4%) had autoimmune HP. The age of the patients at diagnosis varied from 5 to 76 years, being on average 32.9 ± 19 years. The average age at diagnosis varied according to the etiologies: 40.4 ± 16.6 years for post-surgical HP, 15 ± 5.2 years for pseudo-HP, and 12.1 ± 7.4 years for those with other causes-HP. The mean duration of disease was 11.2 ± 7.5 years. Ninety-two percent of the patients were taking calcium supplements with a mean dose of 1,232 mg (range 500-2,150), 80% were receiving daily calcitriol in a mean dose of 0.67 ucg (range: 0.25-2), and 75% were taking cholecalciferol in a mean weekly dose of 35,000 UI (range: 7,000-70,000).

**Table 1:** Demographics of patient cohort

	N = 55
Age (years)	44.5 ± 19.3
Gender	
Female	42 (76.4%)
Male	13 (23.6%)
Age of the onset of hypoparathyroidism	32.9 ± 19.0 (5-76)
Duration of hypoparathyroidism	11.2 ± 7.5 (1-32)
Etiology	
Postsurgical	41 (74.5%)
Autoimune	9 (16.4%)
Pseudohypoparathyroidism	5 (9.1%)

Data are presented as mean ± SD (range) for age at onset and duration of hypoparathyiroidism. Data are presented as N (percent) for the subgroups.

**Figure 1:**
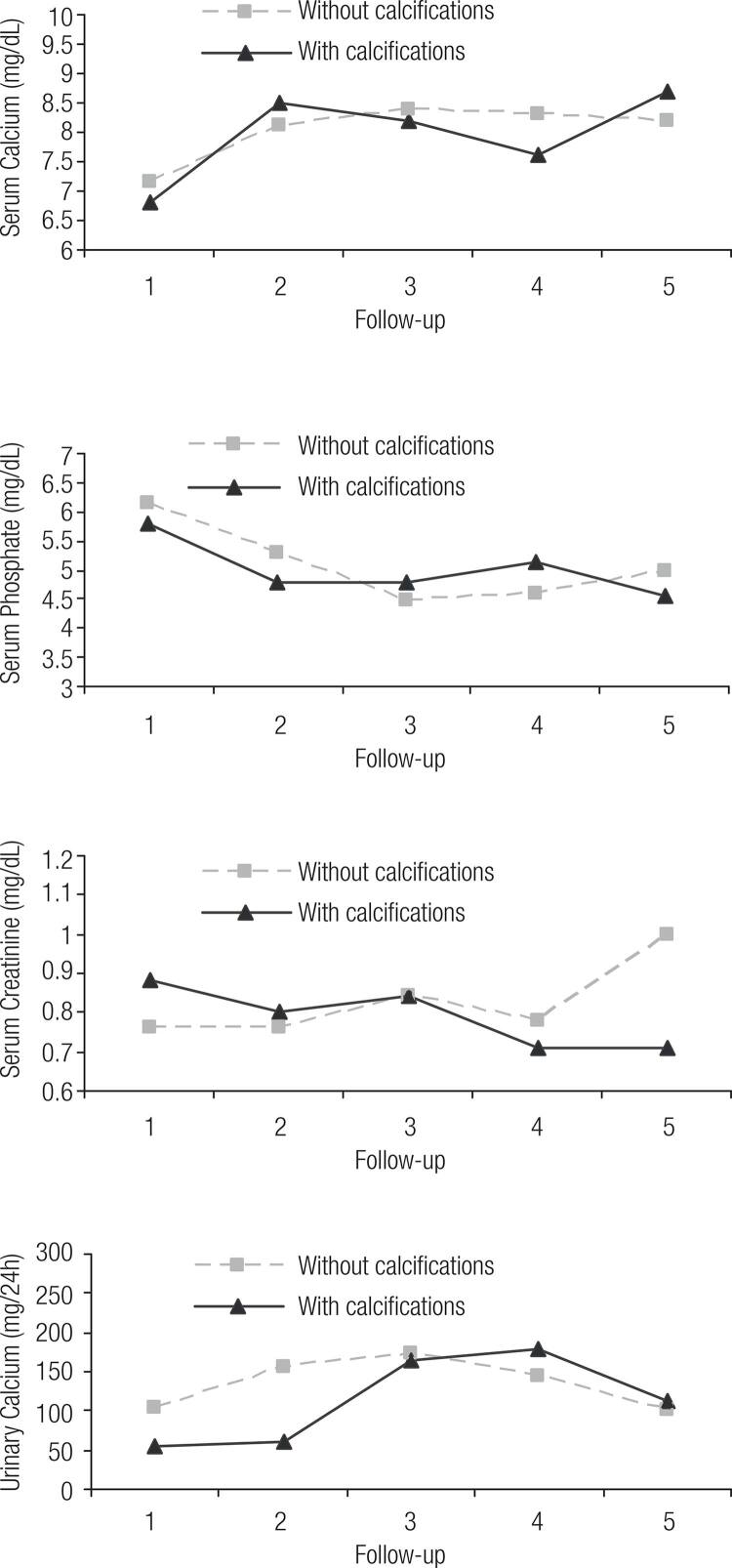
Follow-up of laboratorial findings according to the presence of renal calcification.

Out of the 55 patients, 40 (72.7%) had been submitted to renal USG, whereas 30 (75%) had a normal USG and 10 (25%) had abnormalities such as nephrolithiasis and nephrocalcinosis. No correlation between serum and urinary levels of calcium and the presence of calcification was found. However, weight-adjusted urinary calcium excretion in 24 h was higher in patients with renal calcification than in those without calcification (3.3 vs 1.8 mg/kg/d, p < 0.05). Mean values of serum calcium, phosphate and creatinine, and 24-h urinary calcium in each of the five moments are shown in Figure 1, according to the USG results. The values of serum calcium rose significantly from the first to the last visit (6.87 ± 1.65 to 8.62 ± 1.3; p < 0.001) in the same way that serum phosphate fell significantly (6.14 ± 2.1 to 4.89 ± 1.0; p < 0,001). An inverse correlation was observed between serum calcium and time of disease across different patients, along with a positive correlation with the serum phosphate (R = -0.32, p = 0.033 and R = +0.38, p = 0.013, respectively). No correlation was found between disease duration and levels of creatinine, PTH, or 24-h urinary calcium.

Considering all patients, creatinine values increased during this period (0.81 ± 0.17 – 1.1 ± 0.45), also with statistical significance (p = 0.04). The mean of PTH for patients with pseudo-HP was 130 ± 146 pg/mL; and for the others, PTH was 7.8 ± 11 pg/mL.

**Figure 2:**
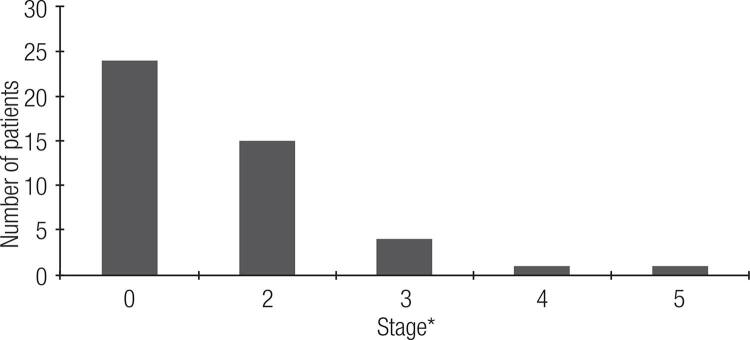
Distribution of patients according their renal failure stage.

Hypercalciuria was found in 15 patients (urinary calcium > 250 mg/24h for females and > 300 mg/24h for males) (16). The glomerular filtration rate (eGFR) varied from 13.8 to 223 mL/min/1.73 m^2^, and its mean was 92.9 ± 36.2 mL/min/1.73 m^2^. Consequently, the renal function was in 15 (33.3%) patients in stage II of chronic kidney disease as stated in Kidney Disease Outcomes Quality Initiative (KDIGO) (17), 4 (8.9%) in stage III, 1 (2.2%) in stage IV, and 1 (2.2%) in stage V (Figure 2). Hence, 21 (46.7%) showed an eGFR lower than 90 mL/min/1.73 m^2^ and would present a chronic kidney disease at least at stage II.

* Stage 1: glomerular filtration rate (GFR) > 90 mL/min/1.73 m^2^; Stage 2: GFR 60-80;

Stage 3: GFR 30-59; Stage 4: 15-29; Stage 5: < 15 or dialysis.

Eleven patients (20%) had a cranial CT evaluation in their records, while 5 (45%) had basal ganglia calcification (BGC). From these patients with BCG, 2 had post-surgical HP, 1 had pseudo-HP, and 2 had HP caused by other etiologies.

## DISCUSSION

This is a pioneer study in our country on hypoparathyroidism (HP) patients followed at a reference center describing the etiologies, laboratory findings, doses of calcium and vitamin D, and complications. In this cohort there was a wide range of age at diagnosis and a high proportion of females, with the post-surgical being the most frequent etiology of the disease (12,18), agreeing with the literature (5,19,20). The most significant clinical feature was the presence of renal complication in this cohort of patients. Kidney calcification and renal function impairment were present in a great number of patients; however, our patients did not have hypercalciuria and the calcium level was within the target range. Kidney calcification and renal function impairment were present in a great number of patients even though mean serum and urinary calcium levels were within the target range. However, higher levels of urinary calcium excretion were seen in patients with renal calcification, suggesting that a upper limit of 4.0 mg/kg/d of urinary calcium (normal range) might not be appropriate for HP patients. Indeed, lower levels of urinary calcium should be the goal for these patients in order to prevent renal complications. Mitchell and cols. reported these findings previously in a cohort of 120 patients with hypoparathyroidism whereas the prevalence of renal calcification was 31% (9). In contrast to our study, they demonstrated a correlation between the duration of the disease and the presence of renal impairment (9). Levy and cols. reported a prevalence of 38% of nephrocalcinosis in children with HP, with the most significant predictors being the degree of relative hypercalcemia and hyperphosphatemia (21). In the present study, no predictor of renal complication was identified such as the doses of calcium and vitamin D supplementation and laboratory abnormalities. This could demonstrate a higher susceptibility some patients may have to developing these calcifications, even when receiving similar doses of treatment as others, or could be the result of the great variability in the serum calcium and phosphorus during the treatment. However, our patients went through a significant worsening of kidney function during the follow-up, despite their younger age. Similarly, Mitchell and cols. found a higher prevalence of eGFR impairment, even though their patients kept serum calcium within the recommended ranges for 86% of the time (9).

In order to avoid these complications, maintenance of serum calcium around the lower limit and serum phosphate around the upper limit of normal ranges is recommended (1,5). In addition, the annual measurement of 24-hour urine calcium is encouraging for ruling out hypercalciuria, as well as renal ultrasound to diagnose nephrolitiases and nephrocalcinosis (5,13). However, it is a challenge to achieve these therapeutic goals, maintaining patients without symptoms, since HP is a chronic disease, and requires long life treatment. Recently, PTH 1-84 has been used in HP treatment in some countries (11,22). This new treatment may lead to control of calcemia without the exposure to high loads of calcium, and therefore may reduce urinary calcium excretion and consequently decrease the incidence of renal complications. Furthermore, this treatment may improve quality of life, an issue that has been discussed in the literature (22,23).

Basal ganglia calcification (BGC) has been established as a possible outcome of HP. Its prevalence demonstrated in the HP cohorts varied significantly from 12% up to 74%. However, it should be noted that the estimated prevalence of BGC in the general population may achieve 12.5% (24). Some studies have shown BGC to be associated more specifically with the persistent high phosphate levels (9,12,24). Contrasting with kidney calcifications, BGC is not a silent outcome of the disease and could be seen at the diagnosis, and thus the cranial CTs may have been performed in patients who already presented neurological symptomatology, leading to the high percentage of our patients with BGC (45%) among those with CT exams. Mitchell and cols., in the same aforementioned study, also found a small percentage (26%) of patients with cranial CT, along with a high percentage (52%) with the presence of BGC (9). Although post-surgical HP is described as rarely causing this complication, because the diagnosis is made early in post-operative care (7), this was the etiology responsible for HP in two out of our five patients with BGC.

In summary, this study obtained data of HP patients followed up in a tertiary care hospital of Curitiba, in the South of Brazil. In this casuistic, it was observed that the treatment led to laboratorial control of calcium and phosphate serum levels. However, the treatment did not avoid an increase in serum creatinine levels and the presence of kidney calcifications. Higher urinary calcium excretion, even if still within the normal range, was associated with development of calcification. Since HP is most commonly a life-long disease, a complete kidney function evaluation, and the search for calcifications is highly recommended. In addition, lower rates of urinary calcium excretion should be aimed for in these patients.
